# Gaps in studies of global health education: an empirical literature review

**DOI:** 10.3402/gha.v8.25709

**Published:** 2015-04-21

**Authors:** Yan Liu, Ying Zhang, Zhaolan Liu, JianLi Wang

**Affiliations:** 1 Department of Psychiatry, Faculty of Medicine, University of Calgary, Calgary, Canada; 2 Department of Community Health Sciences, Faculty of Medicine, University of Calgary, Calgary, Canada; 3 School of Public Health, University of Sydney, Sydney, Australia; 4 Centre for Evidence-Based Chinese Medicine, Beijing University of Chinese Medicine, Beijing, China

**Keywords:** global health, education, gap

## Abstract

**Background:**

Global health has stimulated a lot of students and has attracted the interest of many faculties, thereby initiating the establishment of many academic programs on global health research and education. global health education reflects the increasing attention toward social accountability in medical education.

**Objective:**

This study aims to identify gaps in the studies on global health education.

**Design:**

A critical literature review of empirical studies was conducted using Boolean search techniques.

**Results:**

A total of 238 articles, including 16 reviews, were identified. There had been a boom in the numbers of studies on global health education since 2010. Four gaps were summarized. First, 94.6% of all studies on global health education were conducted in North American and European countries, of which 65.6% were carried out in the United States, followed by Canada (14.3%) and the United Kingdom (9.2%). Only seven studies (2.9%) were conducted in Asian countries, five (2.1%) in Oceania, and two (0.8%) in South American/Caribbean countries. A total of 154 studies (64.4%) were qualitative studies and 64 studies (26.8%) were quantitative studies. Second, elective courses and training or programs were the most frequently used approach for global health education. Third, there was a gap in the standardization of global health education. Finally, it was mainly targeted at medical students, residents, and doctors. It had not granted the demands for global health education of all students majoring in medicine-related studies.

**Conclusions:**

Global health education would be a potentially influential tool for achieving health equity, reducing health disparities, and also for future professional careers. It is the time to build and expand education in global health, especially among developing countries. Global health education should be integrated into primary medical education. Interdisciplinary approaches and interprofessional collaboration were recommended. Collaboration and support from developed countries in global health education should be advocated to narrow the gap and to create further mutual benefits.

The discipline of global health (termed as ‘international health’ previously) has evolved over about 150 years since the cholera outbreak during the mid-1800s (1). Over the past decades, globalization of all aspects of society, including business, media, and education, has been expedited and facilitated by the revolution of Internet/computer (1). Accordingly, global health has become a topic that has drawn considerable attentions.

## Definition of global health

There are still debates about the definition of global health. Without an established definition, it might obscure important differences in philosophy, strategies, and priorities for actions among physicians, researchers, funders, media, and even general public (2).

Many researchers have tried to define global health. Kickbush defines global health as ‘those health issues that transcend national boundaries and governments and call for actions on the global forces that determine the health of people’ (3). Koplan and his collaborators have defined it as ‘the area of study, research and practice that places a priority on improving health and achieving equity in health for all people worldwide’ (2). Beaglehole and Bonita define global health as ‘collaborative trans-national research and action for promoting health for all’ (4) with a clearer, shorter, and sharper emphasis.

Thus, global health is about worldwide health improvement, reduction of disparities, and protection against global threats that disregard national borders (5). Although the generalization of global health varies across intuitions, ‘health for all’ and ‘health equity’ are the main goals of global health (6). The lack of generalization of global health has resulted in the same issue for global health education. Besides, there is no definition of global health education so far.

## Transition of global health

As a result of the union between public health and medicine, the term global health emerged from ‘international health’. International health was already popularly used in the late-19th and early-20th centuries, referring primarily to a focus on the control of epidemics across the boundaries between nations (i.e. ‘international’) (7). Around the mid-20th century, international health grew as an activity to set up health intervention within a broader health system nationally and internationally, aiming to prevent the transmission of infectious diseases (5). Global health implies consideration of the health needs of the people of the whole planet above the concerns of particular nations. Between 1948 and 1998, WHO had moved from being the leader of international health to refashion itself as the coordinator, planner, and leader of global health initiatives (GHIs) (7). WHO did not invent ‘global health’. However, in the 1990s, WHO attempted to use leadership of an emerging concern with ‘global health’. Accordingly, in the 1950s, the number of articles retrieved from PubMed with ‘International Health’ and ‘Global Health’ was 1,007 and 54, respectively. In the 1990s, it was 49,148 and 27,794, respectively. It had been 52,169 and 39,759, respectively, during the period of 2000–July 2005 (7). Now there is an increasing frequency of references to global health.

Based on the logical history of global health, if we want to study global health education, international health education must be considered accordingly.

## Academic organizations on global health research and education

Global health has provoked the interest of a lot of media, students, and faculties and has driven the establishment of several academic programs on global health research and education (2). The Institute for Global Health at the University of California, San Francisco, established in 1999, was the first academic institute to incorporate the term global heath in its name (5). Since then, global health education initiatives and programs were formed quickly, especially in North America (5).

With the development of global health education initiatives and programs, new forms of union and organization are established. Besides WHO, UNICEF, and World Bank, many specialty professional organizations have global health subcommittees. The global health education Consortium, established in 1991, now has a membership of approximately 80 medical schools in the US and Canada and aims to foster global health education for medical students (8). The Global Health Action Committee of the American Medical Student Association was established in 1997 and the International Federation of Medical Students Association (IFMSA) in 1998 (1). Consortium of Universities for Global Health (9), built in 2008, has included 203 university members across the world, including the US, Ethiopia, Pakistan, Egypt, Australia, Israel, India, Mexico, Tanzania, Canada, UK, Japan, Jamaica, etc. Global health education reflects the increasing attention to social accountability in medical education.

## Approaches to global health education

There is no consensus on the approaches to global health education, because medical schools had developed global health curricula independent from each other (10). Most major medical schools are developing global health programs, largely on the basis of resident demands. The vision for a medical school residency program in global health ranged from establishing overseas rotations to developing didactic experiences, experimental experiences, and even incorporating master’s degrees or fellowships into the curriculum (1). Many global health programs simply involved rotations at one or more international sites. A wide variety of programs offered a varied curriculum in both international/abroad and local global health-related experiences (1).

Global health education programs provide different types of fieldwork projects, including epidemiological research, community health, and clinical electives (11). It has the potential to engage students, scholars, and practitioners in ways that go beyond the classroom teaching routine (12).

Digital media technologies might provide feasible and cost-effective alternatives to traditional classroom instruction. However, many emerging global health academic programs lagged behind in the utilization of modern technologies (13).

There are also online programs. As a result, the percentage of pediatric residency programs with information on global health doubled from 23.8% in 2007 to 46.4% in 2009 (14). Digital technologies and online education approaches could simplify and accelerate global health education. However, they would not completely replace the traditional face-to-face interaction teaching and learning experience, especially the fieldwork experience.

## Objective

The aim of this study is to identify gaps in global health education.

## Methods

### Critical review

Relevant qualitative and quantitative articles on global health education were identified from Web of Science (including Web of Science™ Core Collection, KCI-Korean Journal Database, MEDLINE, BIOSIS previews, and SciELO Citation Index, contents of which include regional journals from Latin America and the Caribbean as well as titles from Spain, Portugal, and South Africa) and PubMed. We carried out Boolean search techniques on January 5, 2015, for example, using the combination of key words of (global health* OR international health* OR world health*) AND (education* OR learning* OR training* OR experience* OR teach* OR universit* OR college* OR elective* OR curriculum*), covering the period from 1987 to 2015.

The results of the searches were limited to humans. Searches were performed without language restriction in titles and abstracts in each database. References from retrieved articles were also reviewed for potential applicable publications. Articles and reviews were included by refining document types. However, letters, editorials, meetings, and so on were excluded. Articles dealing with only global health research rather global health education were also excluded. Titles and abstracts of all articles obtained from databases were reviewed.

Information related to global health education was extracted for the retained articles by manually screening, focusing on publishing information, education approaches, standardization, and covering objects to find out the disparity in global health education.

### Articles selecting flowchart

**Fig. 1:**
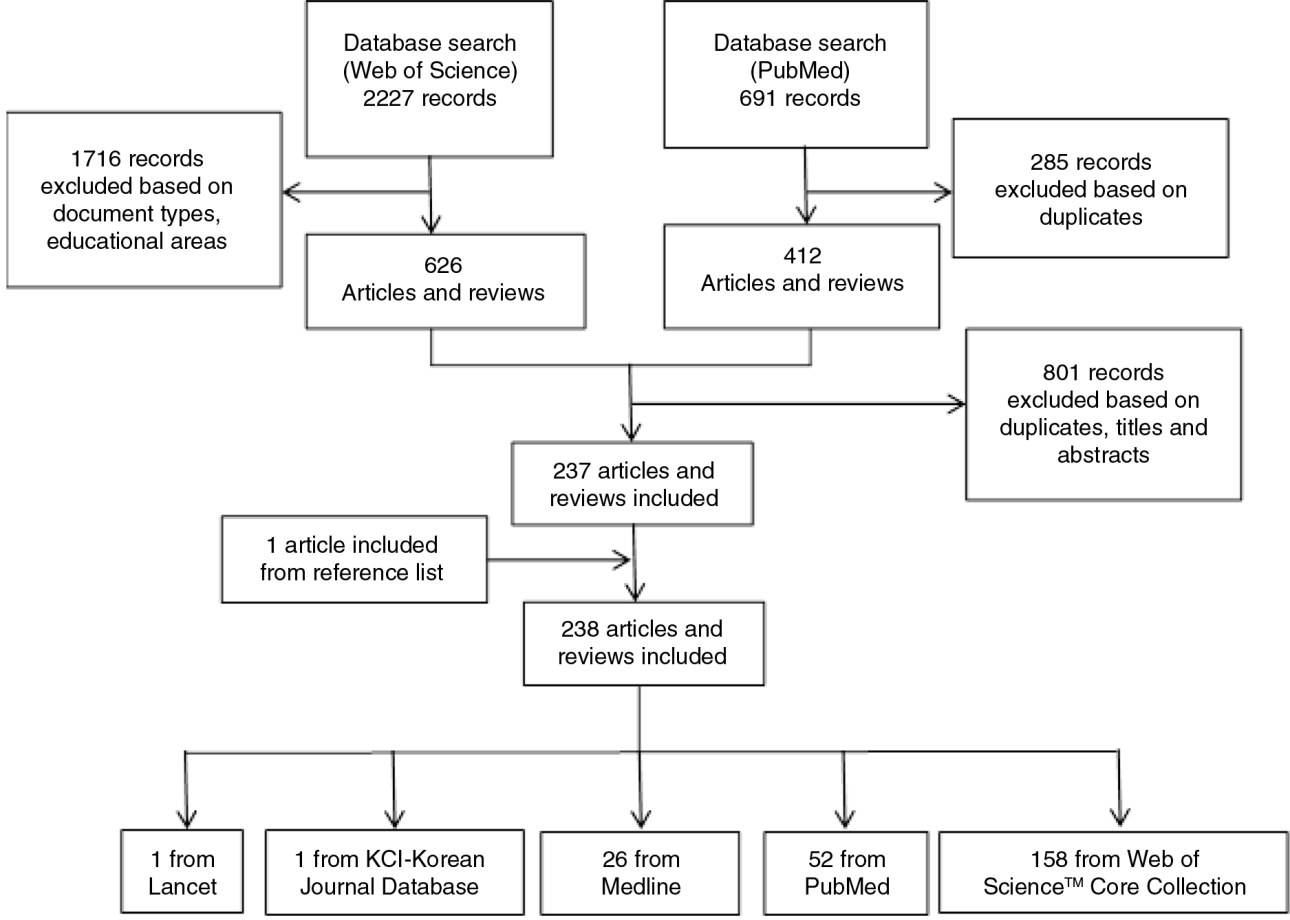
Process of articles selecting.

Figure 1 shows the articles selecting flowchart.

## Results

A total of 238 articles, including 16 reviews (6.7%), were selected in this study. The overviews of all articles were displayed in detail as in Supplementary Table 1. Four gaps in global health education were summarized.

### Trend of studies on global health education

**Fig. 2:**
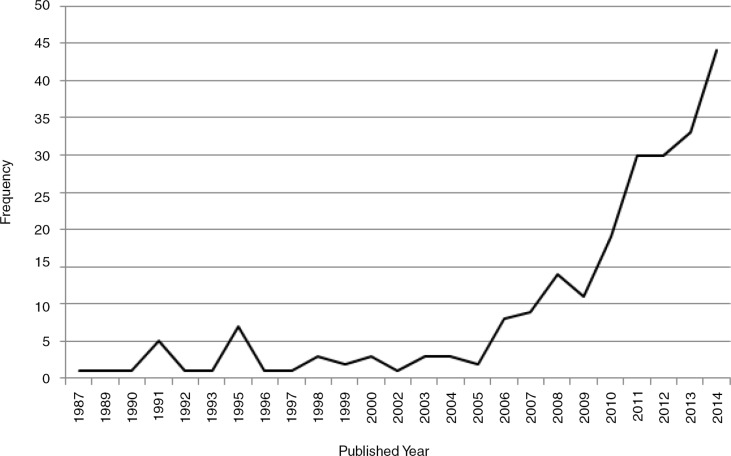
Number of studies on global health education by published year (1987–2014).

Figure 2 shows the trend of studies on global health education. During the past decades, there was no obvious improvement in research on global health education in quantity. During 1987–2007, there were only 53 articles on global health education, with an average of three per year. However, there had been a boom in the numbers of studies on global health education since 2010. The numbers of articles addressing global health education in 2011, 2012, 2013, and 2014 were 30, 30, 33, and 44, respectively.

### Gap in study quantity and quality in global health education

The first obvious gap was in the quantity of studies on global health education between developed countries and developing countries. A major part of the of the studies on global health education (94.6%) were conducted in North American and European countries, of which 65.6% were carried out in the United States, followed by Canada (14.3%), and the United Kingdom (9.2%). Only seven studies (2.9%) were conducted in Asian countries, five (2.1%) in Oceania countries, and two (0.8%) in South American/Caribbean countries (Table 1). No study has been done in African countries as yet. However, many developing countries, such as Uganda, Botswana, Kenya, India, Thailand, and Mexico, have partnered with developed countries to offer international experience for trainees in their global health education.

**Table 1:** Percentage of studies on global health education (GHE) by region

Region	Nation	Number of studies on GHE	%
North American	USA	156	65.5
	Canada	34	14.3
	Total	191	80.3
European	UK	22	9.2
	Italy	3	1.3
	Hungary	1	0.4
	Denmark	2	0.8
	Netherlands	1	0.4
	Ireland	1	0.4
	Russia	1	0.4
	Germany	3	1.3
	Total	34	14.3
Oceania	Australia	4	1.7
	New Zealand	1	0.4
	Total	5	2.1
South America/	Mexico	1	0.4
The Caribbean	Jamaica	1	0.4
	Total	2	0.8
Asian	Israel	2	0.8
	Japan	3	1.3
	South Korea	1	0.4
	India	1	0.4
	Total	7	2.9
Total		238	100.0

In terms of study quality, 154 studies (64.4%) were qualitative studies and 64 studies (26.8%) were quantitative studies. Five studies had used mixed qualitative and quantitative methods. The sample size ranged from 1 to 1,126. Only 33 articles had a sample size over 100.

### Gap in education approaches in global health education

Although global health education was so prevalent, it varied in contents and approaches. Most of the studies showed that electives’ curriculum/course/disciplines were the most frequently used approach (which was mentioned in 80 articles) for global health education. Global health training/programs/fellowship was also mentioned in 65 articles. There were four articles referred to master’s program (5, 15–17), one article involved the Bachelor of Science in International Health (18) and one related to Certificate in Global Health (19). All details are displayed in Supplementary Table 1.

In terms of teaching methods, not only didactics and experiential experiences (10, 20), research-based narrative assignment (21), field-based experience (domestic and abroad training/rotation) (22–25) were discussed, distance learning/e-learning/web-based learning (17, 26–29), global health–related digital media products (13) was also recommended. Furthermore, transdisciplinary approach and interprofessional collaboration were mentioned in seven articles (30–36).

In terms of training period, the shortest period was 1 week (25, 37) and the longest one was 2 years (38), with the average time slot from 4 to 8 weeks.

No study was focused on the comparison of effects among different approaches. This would be a new theme for future research.

### Gap in standardization of global health education

University/medical schools had developed global health curricula independent from each other (10). This had resulted in the third gap, that is, a lack of standardization of global health education, such as the standardization of curriculum, approaches, and programs. For example, global health centers had been established at Harvard University (39, 40) and Johns Hopkins (28) to offer global health education. However, Duke University had taken up to offer a Master of Science in Global Health (41), which was one of the first programs of this kind in the United States. Beginning fall 2014, Duke will also offer the program at Duke Kunshan University in China (42). Several universities in Australia also offer a Master Degree on international health, for example, the Master of International Public Health for students at the University of Sydney (43), which could be completed fully online.

Despite the increased interest by resident trainees, little has been done in the standardization of these experiences (44). Program directors were responsible for identifying the educational merit of global health electives without having first-hand knowledge of the experience. Without standardization, large disparities remained in funding, accreditation, oversight, and evaluation among global health training programs (45).

### Gap in covering objects in global health education

The fourth gap was about education covering objects. Current global health education programs and curricula were mainly targeted on medical students (73 articles referred) and residents (including pediatrics, otolaryngology, obstetrics and gynecology, emergency medicine, surgery, radiology, psychiatry) (53 articles referred). Some were focused on public health or international health students (16 articles referred) and nurse/nursing students (14 articles). Ten articles had mentioned doctors/physicians. Meanwhile, among the 156 studies conducted in the United States, 111 articles were targeted on medical students, residents, and clinical doctors. Only 19 articles were focused on public health or international health students and nursing students.

Furthermore, undergraduate students were mentioned in 12 articles and graduate/postgraduate students were mentioned in eight articles. Master students and PhD students were also mentioned in five articles.

The deficit covering also caused another gap: the demands for global health education among students or residents and the provisions of global health Education courses from universities in developed countries. There was an obvious lack of multidisciplinary approach that could be attractive to other disciplines associated with global health, for example, that of pharmacist and stomatologist. In Japan, a national survey of 150 members of the Japan Association of the Directions of the Departments of Hygiene and Public Health at 80 medical schools revealed that only 40.7% of the departments offered international health curriculum (45, 46). There was no study on student demands for global health education in developing countries.

This was a new topic about global health education deserving attention and further research: should it be essential or possible that global health education would be offered to all students (including undergraduate and graduate students) whose major was medical related?

## Discussion

### Discussion of findings

Although global health education is gaining more and more attention, there was still a lack of consensus on the contents and approaches, and there were mainly four gaps in global health education: researches between developed and developing countries, education approaches, standardization of global health education, the targeted objects and student demands and institution/university offering.

With the awareness of the development of global health, many academic programs on global health trainings have emerged and are well established, particularly in North America (47). Obviously, the United States is taking a leadership role in the effort to improve global health now. Macfarlane and his colleagues’ study showed that 87% of global health institutions were located in North America in 2008 (5). The United States has global health investments and programs in approximately 80 countries worldwide, and all of these countries will be included in GHI, established in 2009. More and more medial students or residents have been exposed to global health education. In 2001, 20% of graduating US medical students reported having participated in a global health training experience (48), up from 5% in 1984 (49). This number had increased to 30.5% in 2011 (50).

One of the most feasible reasons for the leading position of the United States is that global health education has been initiated by the United States and has been imitated by other countries and regions outside North America, such as Australia (Deakin, Melbourne, and Sydney Universities), UK (University College London, University of Oxford, etc.), Norway (University of Bergen), Ireland (University of Dublin), Sweden (Lund and Umeå Universities), Japan (Universities of Hokkaido, Okayama, Ryukyus, Kyoto, and Tokyo), Brazil (Ceará Federal University), Kenya (Kenya Medical Research Institute), China (Peking, Fudan Universities), and Israel (Ben-Gurion University) (5), which have offered global health education courses or programs. Besides the academic global health institutions, national networks were also formed by academic institutions, scientific societies, nongovernmental organizations, associations, groups and individuals engaging teaching global health, Such as the Italian Network for Education on Global Health (RIISG) which was created in 2007 with the purpose of spreading the concept of GH (51). Furthermore, global health education in North America is constantly updated representing a paradigm shift in structure and function, aiming to train future global health leaders (31). This should be the most important learning experience for developing counties to develop their own national global health education.

Unfortunately, we found that there were inadequate published researches in developing countries, especially in Asian and African regions. This did not mean there were no global health education activities in these regions but there were no enough publications on global health education. This generated a publishing bias. Take China as an example, the PKU-DUKE Global Health Certificate Program (52) is held in Peking University each summer initiated from 2009. Each year, it offered a 2-week global health education to over 30 students or professional faculties from all over China. This program had proceeded for 6 years. However, there were still no published studies about this program. This prompts us that it is time for developing countries to take complete and speedy researches on global health education based on the abundant experience obtained from developed countries.

The promotion of global health education should not be confined by traditional approaches (e.g. electives, programs, certificate education). Establishing of thought-out educational approaches should be prerequisites for international or interinstitutional cooperation. The current environment of somewhat fragmented curricular development is gradually transiting to increased collaboration, emergence of best practices and shared models. Interdisciplinary approaches and interprofessional collaboration were recommended (30–36). Here was a typical example of a layered model. The global health curriculum at the University of Vermont College of Medicine provided a baseline level of global health education to all medical students, via introductory lectures at Orientation, matching with a global health-oriented faculty member on request and a Bridge curriculum in global health between the clerkship and senior year of medical school. A didactic 1-month elective in global health was available to all senior students, as it was a 1-month abroad elective at one of two partner sites in Bangladesh, with an equal emphasis on development of the host center (53). Each year, approximately 15–20 students (18–25% of the class) opted for the didactic elective, and 3–5 for the experiential abroad elective. For the past 3 years, at least one student had pursued an MPH degree for further training in global and community health each year (53).

Global health education could be a potentially influential tool for achieving health equity, reducing health disparities and also for future professional careers. However, there has been an argument that all medical students should be exposed to global health education. All health professionals, regardless of their location and specialty, practice independently. It is therefore the responsibility of educational institutions, educators, and students to ensure that physicians/residents are well equipped for the complex challenges in the coming decades (54). Global health represents exciting opportunities for teachers and learners alike. As medical professionals engage further with global health and with counterparts, there is an opportunity for shared learning (55). Various groups have been working on addressing the perceived gaps in global health education by proposing global health competencies for undergraduate medical students (56–58). Actually, not only medical students (undergraduate and postgraduate included) but also students of public health, nursing, nutrition, law, and so on, should acquaint themselves with global health.

Although there is no common understanding of the definition, contents, approached and education objects, and even no obvious trend for developing countries to develop academic global health education (5), there has been a growing interest in cross-cultural collaborations and educational initiatives, with the purpose to enrich the experiences of health professionals, and to improve the health globally. Collaboration and support from developed countries in global health education should be advocated to narrow the gap and create mutual benefits. The future of the global health requires partnerships among nations, health care professionals, medical researchers, public health specialists, corporations, and individuals. As WHO Macroeconomic Commission on Health has reported that we have the ability and technology to save millions of lives each year if only the wealthier countries would provide poorer countries with such health care and services help (59).

Here comes a good example of collaboration on global health education development: the Association of Pacific Rim Universities (60), formed in 1997, consisting of 45 leading research universities, from countries such as Australia, Canada, Chile, China, Russia, the US, and so on, aiming to foster education, research, and enterprise in the Pacific Rim. From 2012, APRU has embarked on a new strategic framework including creating Asia-Pacific Global Leaders. APRU universities will cooperate to enhance the global leadership capabilities of faculty, administrators, and students, as well as of their institutions.

In this study, the gap between demands and provision was mainly based on students’ perception. However, students’ perception alone is not enough to decide about the GHE, perception of curriculum committees and educators involved in teaching global health should also be included. Although 83% of Psychiatry Residency Training Directors respondents (as a total of 59) thought global health education were important in professional development and cultural exposure, obstacles including lack of accreditation, financial resources, and faculty or administrative support and supervision had made it unavailable (61). Anyway, to close the gap between education demands and education offering on global health, global health education should be integrated into primary medical education through health policy legislation and education of medical students (62, 63). The benefits of integrating global health education into primary medical education are significant (56). On one hand, integration ensures that the medical students as a whole have access to the global health education that they need early with the background of globalization. On the other hand, when medical students receive global health education in primary medical education, the likelihood of wider health view and health practices, as well as maintained social integration will be increased (56).

In terms of future global health education, institutions developing or evaluating global health education programs should focus on the following themes (64): the definition and scope of global health education; the contents and approaches of global health education; the standardization of global health education; the demands on global health education of medicine-related students in developing countries; the challenges and opportunities associated with interinstitutional or interprofessional collaborations and the evaluation of global health education.

## Discussion of limitations

There are limitations in this study. First, the databases we searched are limited. Second, during the articles manual screening process, mistakes might have been inevitable. Third, it is really hard to count all academic global health education initiatives and programs because new ones are established so quickly and there are no accredited list records. Therefore, we could only conduct our study by searching published articles on global health education and this might have resulted in information bias.

## Conclusions

Although there are limitations in this study, we still hope to increase the awareness of global health education issues and to empower students from developing countries to contribute to global health in their futures. It is also the time to build and expand education in global health, especially among developing countries. global health education should be integrated into primary medical education. Interdisciplinary approaches and interprofessional collaboration were recommended. Collaboration and support from developed countries for global health education should be advocated to narrow the gap and create further mutual benefits.

In this increasingly globalized world, we believe that such measures: establishing standardization for global health education, expanding the coverage of global health education, and strengthening global cooperation, could help achieve the global health goals of ‘health for all’ and ‘health equity’ and might narrow the global health education gaps in the near future.

## Supplementary Material

Gaps in studies of global health education: an empirical literature review
